# Intriguing Origins of Protein Lysine Methylation: Influencing Cell Function Through Dynamic Methylation

**DOI:** 10.1016/j.gpb.2019.03.005

**Published:** 2020-03-17

**Authors:** Natalie Mezey, William C.S. Cho, Kyle K. Biggar

**Affiliations:** 1 Institute of Biochemistry and Department of Biology, Carleton University, Ottawa, ON K1S 5B6, Canada; 2 Department of Clinical Oncology, Queen Elizabeth Hospital, Kowloon, Hong Kong Special Administrative Region, China

## Introduction

*“There is a kink (shoulder) on [the] Lys peak…”* These words scribed in Richard P. Ambler’s laboratory notebook marked the discovery of protein methylation and a segue into a new field of scientific research [1]. Initially, through ion-exchange chromatography and two-dimensional paper chromatography of the hydrolysate of *Salmonella typhimurium* flagellin, this “kink” was interpreted as a “new amino acid”, the ε-*N*-methyl-lysine (NML). This discovery, bolstered by a subsequent examination of purified NML, was the first glimpse of protein methylation in living cells [1].

Although this exciting new discovery led to an initial surge in interest, focus on protein methylation quickly waned for a number of decades. By the time protein methylation emerged as a field of interest, research into other post-translational modifications (PTMs) was firmly underway. For example, the discovery of lysine (Lys) methylation predated tyrosine (Tyr) phosphorylation by two decades, following a fortuitous discovery of this new type of protein modification in v-Src associated kinase activity [2]. Following the path of early researchers in the discovery of non-histone protein methylation toward modern discoveries in methyllysine proteomics, this article aimed to unpack the key discoveries which paved the way of further understanding and characterization of the functional impact of this small modification over important cellular processes such as cellular growth signaling and DNA damage response, as well as other cellular pathways in disease pathology.

## Post-translational lysine methylation: not just a mere afterthought

Although it was first discovered in 1959, protein methylation has only become a prolific area of discovery in recent decades. What we currently know of its properties and significance in biological function leaves many unanswered questions, making it all the more intriguing as research efforts continue.

PTM is well-known to regulate a wide range of biological functions, including the regulation of numerous protein interactions, protein localization, protein stability, and enzyme function [3]. However, protein lysine methylation has been primarily observed and studied on histone proteins, owing to its size, prevalence, and importance in packaging eukaryotic DNA into chromatin. For example, methylation of Lys 4 of histone H3 (*i.e.*, H3K4me) is a well-established marker of gene activation [4]. Other common sites of methylation that are associated with gene activation include H3K79, whereas sites associated with gene inactivation include H3K9 and H3K27 [4]. In addition, a combination of multiple PTMs (including phosphorylation, acetylation, and ubiquitination) are also involved in the regulation of chromatin structure and gene expression, collectively representing what is known as the “histone code”. Of these PTMs, methylation is the smallest, has little steric bulk, does not affect charge, and can exist as one of three distinct methyl-forms (mono-, di-, and tri-methylation) [5]. Indeed, it has been proposed that the relatively slow kinetics of mono-, di-, and tri-methylation of histone Lys residues contribute to epigenetic stability for histone proteins [6]. Patterns of histone methylation help to regulate chromatin structure and accessibility, while also explaining epigenetic signaling and phenotypic diversity between cell types. As a result, diseases such as cancer and intellectual disability can result from imbalance of these methylation markers [7].

Following the extensive characterization of histone methylation, Lys methylation of non-histone proteins has been recently found to regulate many cellular processes [8]. Lys methylation is a functionally important PTM occurring on histone proteins. Although numerous lysine methyltransferase and demethylase enzymes have been characterized regarding their ability to control methylation at specific histone residues, their known targets have been rapidly expanding to include the methylation of non-histone proteins as well. Collectively, these findings have extended the role of Lys methylation well beyond the established “histone code” and its role in epigenetic regulation. Hundreds of such proteins are methylated at Lys residues and this PTM is involved in regulating cellular growth signaling and DNA damage response [8]. Approximately 80 Lys methyltransferase (KMT) and demethylase (KDM) enzymes have been discovered to regulate Lys methylation, with several displaying specificity only toward non-histone substrates [8], [9]. Despite the number of known Tyr phosphorylation sites (upward of 20,000 modification sites) far outnumbering identified Lys methylation sites, the number of Lys modification sites have been on a continuous increase in recent years as new and reliable identification technologies are developed [10]. Indeed, methylation of non-histone proteins has emerged in recent years as a PTM with wide-ranging cellular implications since its discovery in 1959.

## The 1960s: swinging the pendulum of research in the direction of lysine methylation

Ambler and Rees’ observation of methyllysine in the flagellin of *Salmonella typhimurium* provided the scientific community with proof of protein methylation in living cells [11]. In addition to this pivotal discovery, their research also led them to the discovery of a separate gene, which determined the presence or absence of methyllysine in flagellin, thereby demonstrating that methylation was indeed a PTM, and also providing a subtle hint into its dynamic nature. Ambler and Rees’ research was an impetus for further exploration. They reasoned that a specific enzyme must be responsible for the methylated Lys residues of a protein. These early theories lay the groundwork for the revelations in the field with implications that are still realized today.

Although the physiological and regulatory roles of other PTMs such as phosphorylation had already been explored [12], [13], [14], the 1960s brought important contributions to the most basic understanding of Lys methylation. In 1964, Kenneth Murray discovered the presence of methyllysine in the hydrolysate of histones [15]. Kim and Paik demonstrated that methyllysine could not be conjugated to tRNAs, thus resolving a persisting question on how and when methylation occurred [16]. This discovery confirmed the earlier suggestion that histones were methylated following protein synthesis, not before. Building on these insights, Vincent Allfrey and fellow researchers posited what at the time would have been a truly prescient hypothesis, that methylation of histones could regulate gene transcription [17].

**Figure 1:**
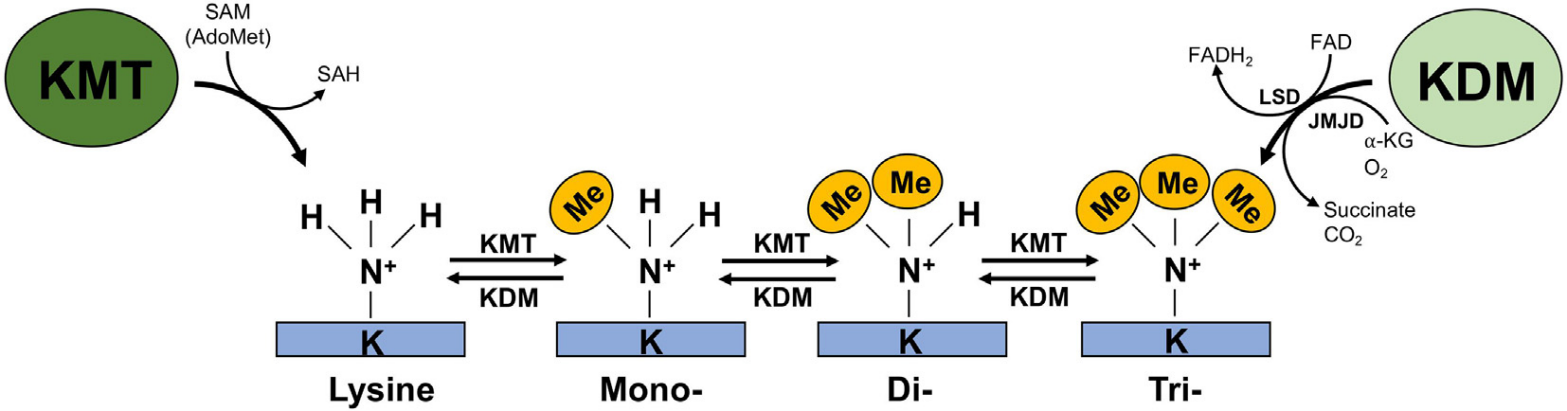
**Status of Lys methylation events** Lys methylation is directed by the opposing actions of lysine methyltransferase (KMT) and demethylase (KDM) enzymes. Three methylation groups can be added by a KMT, or removed by a KDM enzyme, at a single Lys residue resulting in the formation of mono-, di- or tri-methyllysine. KMT enzymes transfer the methyl-group from the *S*-adenosylmethionine (SAM) methyl-donor to a Lys residue, resulting in a methyl-modified Lys and *S*-adenosylhomocysteine (SAH). KDM enzymes demethylase methyllysine residues through one of two known families, lysine-specific demethylases (LSDs) or Jumonji-domain containing demethylases (JMJDs). LSD-regulated demethylation is dependent on flavin adenine dinucleotide (FAD) reduction to FADH2 (hydroquinone form). JMJD demethylases are oxygen (O_2_) and alpha-ketoglutarate (α-KG)-dependent and stoichiometrically produce succinate and carbon dioxide (CO_2_) upon the successful demethylation of substrate.

Following this initial curiosity, there was a precipitous drop in research in subsequent decades, in large part because no causal link could be established between protein methylation and regulation of biological processes. Kim and Paik diverted their focus to the enzymes involved in methylation throughout the 1960s and 70s. This was a fortunate detour, as they were able to establish the first methyltransferase activity: the enzymatic transfer of a methyl group from *S*-adenosylmethionine (SAM) to Lys, Arg, Asp, or Glu residues [16]. In the case of KMTs, it was determined that these enzymes were able to add a maximum of 3 methyl groups to the ε-nitrogen of the Lys residue (Figure 1) [18].

## The 1990s and onwards: a revival of unfinished research

It was not until the 1990s that hints of a functional role for Lys methylation were finally beginning to be uncovered. The progress in genetics and molecular biology were propellers of these advancements, notably the expansion of genomic sequence information and techniques for studying chromatin biology [19]. Starting with Thomas Jenuwein’s gene ablation experiments identifying SUV39H1 as the first Lys-specific histone KMT in 2000 [20], 50 SET domain-containing proteins were subsequently identified as Lys-specific KMTs [21].

In what is referred to as the “histone code”, PTMs of histones (such as methylation) were interpreted as a unique language which spelled out transcription and chromatin structure [20]. Jenuwein et al. further contributed to this knowledge by discovering that these signals were interpreted by reader proteins [22]. Methylated Lys residues are recognized by methyl-binding proteins which “read” the adjacent amino acid sequence and interact with the methylated residue through a conserved aromatic cage, creating a methyl-dependent protein interaction [23]. In addition to interpreting methylated Lys residues on histone tails, these reader proteins also play a regulatory role of different biological mechanisms such as crosstalk and gene transcription. These residues are stabilized through the strong attractive forces of the cation and the negative π-surface of the aromatic ring [24]. Conversely, a non-methylated Lys residue displays acidic residues, thus allowing for reader proteins to be selective based on the ratio of aromatic to acidic residues.

Shortly thereafter, large families of protein methyl-binding domains such as plant homeodomain (PHD) finger-containing domains, Tudor domains, malignant brain tumour (MBT) repeats, chromo domains were added to the list [25]. There was an increasing suspicion that the process of methylation must be reversible. This was confirmed by the discovery of Lys-specific demethylase 1 (LSD1), a KDM that demethylates H3K4me1 and H3K4me2 substrates [26]. LSD1 showed that protein methylation is a dynamic process similar to protein phosphorylation, a view which until that point had been strongly contested.

Zhang and his colleagues added Jumonji C-terminal (JmjC) domain-containing KDMs. JmjC-domain-containing proteins (also known as JMJD) were a class of alpha-ketoglutarate-dependent KDMs, which demonstrated a unique method of methylation. While LSD1 demethylates by oxidizing the ε-amino group of Lys, the larger class of JmjC-domain-containing KDMs oxidize the methyl groups, enabling the demethylation of Lys residues [27]. Taken together, this creates a dynamic writer, reader, and eraser model that is analogous to dynamic Tyr phosphorylation [8]. In this system, KMT functions as a ‘writer’ to add a methyl-moiety to a Lys residue on a given substrate. The chemical change accompanying methylation may also facilitate interactions with ‘reader’ methyl-binding proteins. The methylation signal is then terminated by a KDM, ‘erasing’ the Lys modification and returning the substrate to the demethylated state.

## Moving on from histones: exploration of the non-histone methyl lysine proteome

While the role of Lys methylation in histones had been already being elucidated, the discovery of RNA-binding protein (RBP) methylation in 1998 expanded the scope of protein methylation. The methylation of RBPs was shown to have a regulatory role in RNP assembly, pre-mRNA splicing, and mRNA stability [28].

In 2004, Reinberg and his team observed an important function of Lys methylation in p53 tumor suppressor protein. In particular, they found that methylation of p53 by the KMT, SET domain-containing protein 7 (SETD7), resulted in enhanced transcriptional activity, nuclear stability, as well as apoptosis [29]. Subsequent studies revealed that p53 could function as an activator or repressor in response to dynamic methylation status of four neighboring Lys residues [30]. In 2007, Berger found that p53 could also be demethylated. Specifically, the KDM, LSD1, was found to demethylate the di-methylation modification at K370 (*i.e.*, loss of p53K370me2), thereby disrupting the methyl-reader abilities of the Tudor domains of p53-binding protein 1 (53BP1) [31]. This ultimately resulted in the repression of p53 and DNA damage response. Furthermore, a number of different Lys methylation sites have been documented to be present in the catalytic subunit of DNA-dependent protein kinase (DNA-PKcs). The status of these Lys methylation events are suggested to dictate the ability of DNA-PKcs to effectively repair damaged DNA [32].

Further highlighting the broad reach of KMTs in non-histone Lys methylation, SETD7 has also been found to be the primary KMT for the methylation of ribosomal protein L29 (Rpl29) at K5 (*i.e.*, Rpl29K5), a ribosomal protein that is prevalent in all cell types. Lys methylation of Rpl29 dictates its regulation and impacts subcellular localization. It has been determined that methylation of Rpl29K5 is so frequent that the methylation itself may be used as a cellular biomarker for SETD7 activity. Consequently, it is possible that Rpl29 methylation can be used as a target for SETD7 inhibitors [33]. Like p53, Rpl29 is demethylated by LSD1. LSD1 also demethylates, DNMT1, E2F1, as well as STAT3 [34]. The methylation of transcription factor E2F1 by SETD7 at K185 initiates the DNA damage response pathway by regulating the transcription of genes involved in repair [35].

Ubiquitin-like with PHD and RING finger domains1 (UHRF1) are also methylated by SETD7 and demethylated by LSD1. UHRF1 functions to regulate DNA methylation as well as heterochromatin formation. Methylation of UHRF1 has been shown to have unique functional response to DNA damage by regulating the enzymatic activity of repair proteins or the binding affinity of repair-associated transcription factors. Specifically, the methylation of UHRF1 induces the homologous recombination required for DNA repair, thus playing a critical role in the double-strand break repair mechanism [36].

Despite the rapid growth in our understanding of the function of Lys methylation, the field has experienced limited growth as a result of a lack of suitable identification technologies. Methylation exists as a relatively small uncharged protein modification. As a result, it is difficult to develop antibodies that do not suffer from low affinity or poor specificity, or that do not maintain specificity for the amino acid sequences surrounding the modified Lys. Although there have been several reports of successful immunoaffinity-enrichment of methyllysine peptides [10], [37], a growing interest has emerged in the development of new chromatographic methods of enrichment [38] and the utility of naturally-occurring protein methyl-binding domains for affinity-based purification and enrichment prior to identification by mass spectrometry [32], [39], [40]. As methyllysine-specific antibodies cannot provide information of direct physical interactions that may occur in the cell, the use of methyl-binding domains has been utilized for the mapping of methyl-dependent protein complexes, a collection of interactions referred to as the methyl-interactome [32].

**Figure 2:**
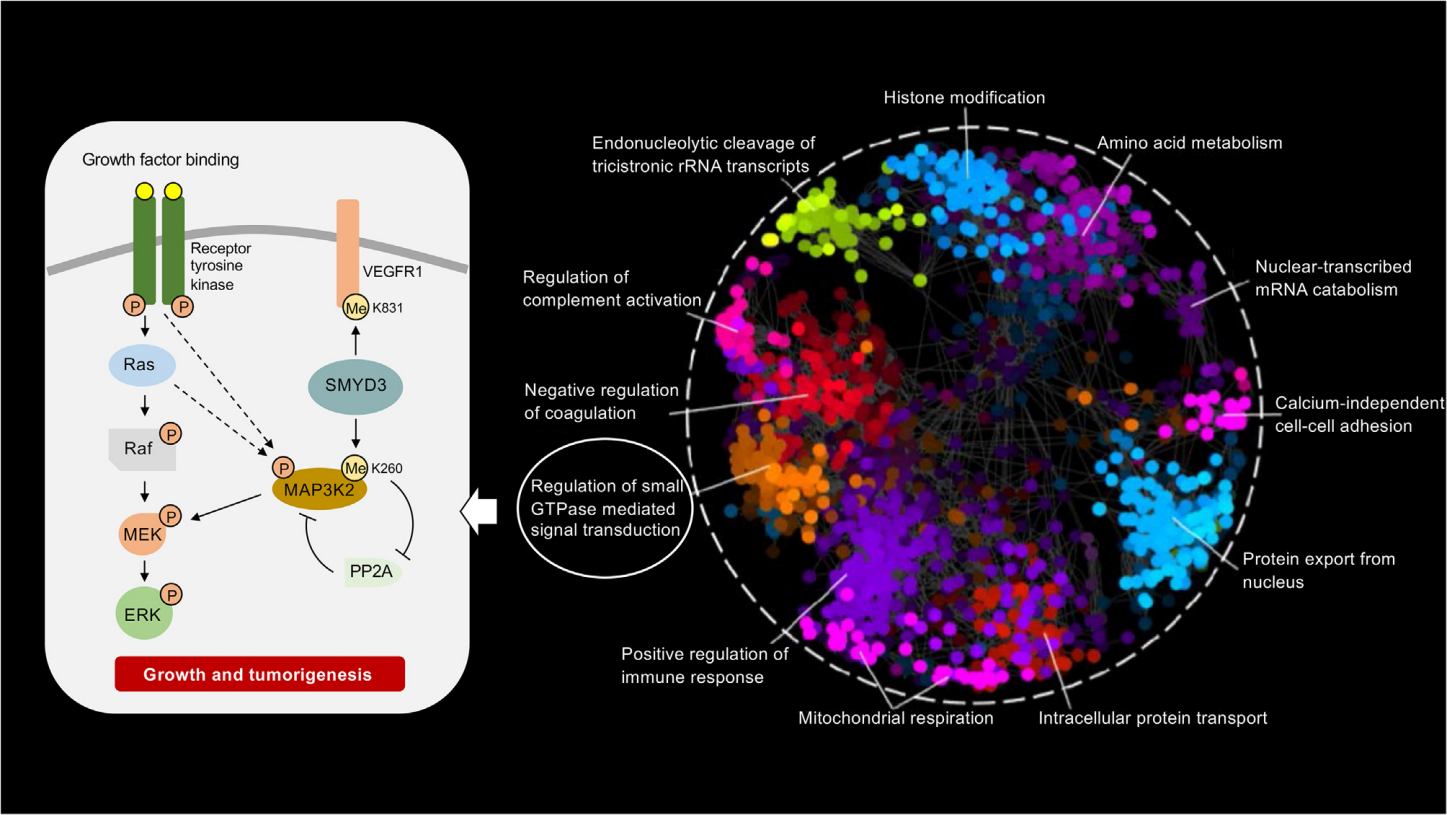
**The functional human methyllysine proteome network** Human Gene Ontology (GO) enrichments of biological processes for the methyllysine proteome (PhosphoSitePlus; accessed 01-02-2019). To obtain a functional annotation of Lys-methylated proteins, a Spatial Analysis of Functional Enrichment was visualized using Cytoscape (v.3.5.1). All clusters are differentially colored and annotated using human GO terms. SMYD3-directed methylations of MAP3K2 at K260 and vascular endothelial growth factor receptor 1 (VEGFR1) at K831 are depicted as functional examples of Lys methylation.

As we expand the breadth of protein Lys methylation events, there is a growing realization that Lys methylation plays a critical role in the development of many human diseases. Given the knowledge that Lys methylation plays a functional role in the regulation of an ever-expanding list of cellular processes (Figure 2), perhaps this is not surprising [41], [42]. One pivotal study discovered that the KMT SET and MYND domain containing 3 (SMYD3) is a driver of Ras-driven leukemia, mediated by SMYD3-dependent methylation of the MAP3K2 protein at K260 (Figure 2) [43]. Given the involvement of Lys methylation in a growing number of different biological processes [8], it is not surprising that methylation has been increasingly documented to be critically important to human health. As modifiers of Lys methylation status, both KMT and KDM enzymes have correspondingly emerged as promising drug targets [18], [44]. For example, SMYD3 is frequently upregulated in human colorectal, liver, and breast cancer cells, compared to their matched non-cancerous cells where expression is nearly undetectable, and this activity is associated with the growth of these tumors [45]. Taken together, these data provide an intriguing insight into how KMT dysfunction plays a crucial role in carcinogenesis. The inhibition of methyl-regulating enzymes could provide a novel therapeutic strategy for treatment of not only breast cancer, but also for the treatment of other cancers where KMT and KDM enzymes are involved.

## Targeting lysine methylation for drug development

Targeted therapies are not available for a number of cancers. For example, systemic chemotherapy is the only treatment option for triple negative breast cancer after surgery. However, chemotherapy is highly toxic and cancer cells can eventually become resistant to the treatment. New drug targets and innovative research strategies are key for the cancer therapeutics. Recently, research in cancer biology has discovered that genes encoding KMT and KDM enzymes, *e.g.*, the KMT2 (MLL) family proteins, are collectively among the most frequently dysregulated genes in many types of human cancers, and there is now a strong interest in developing targeted therapies against these modifying enzymes.

Given the extensive regulatory importance realized for Lys methylation, any mutations or dysfunction in KMT or KDM enzymes can lead to deregulated cell function, tumorigenesis, and chemotherapy resistance [46], [47]. Indeed, a number of high-quality inhibitors for a handful of these enzymes have been recently identified. Several of these inhibitors elicit selective cancer killing *in vitro* and robust efficacy *in vivo*, suggesting that targeting Lys methylation pathways and their regulating enzymes may be a relevant, emerging cancer therapeutic strategy.

To date, a handful of KMT and KDM inhibitors have been discovered or developed, still many inhibitors are at the preclinical stages of development (Table 1) [47]. Indeed, given the similarity between catalytic domains among families of KMT and KDM enzymes, it has been difficult to develop an inhibitor specific for a dysfunctional enzyme without significant off-target effects. For example, the demethylase enzyme, LSD1, has been identified as a high-priority drug target as it has been found to be over-expressed in several different types of human cancer, playing a crucial role in cancer cell growth and proliferation. However, due to the similarity among catalytic sites and structural features, drugs targeting LSD1 have also been reported to act as monoamine oxidase (MAO) inhibitors [48]. At this point, several potent small molecule inhibitors of LSD1 have been discovered and show inhibitory activities *in vitro* and *in vivo* on various cancer cells.

**Table 1:** **Examples of KMT inhibitors that are under development and therapeutic testing**

**Compound**	**Target**	**Cellular potency (IC_50_)**	***In vivo* activity**	**Status**	**Ref.**
BIX-01294	EHMT1/2	500 ± 43 nM	No	Preclinical	[58]
UNC0638	EHMT1/2	81 ± 9 nM	No	Biological testing	[59]
EPZ005687	EZH2	80 ± 30 nM	No	Preclinical	[60]
EPZ6438 (tazemetostat)	EZH2	8 nM	Yes	Phase II	[61]
GSK126	EZH2	28 nM	Yes	Phase I	[62]
GSK343	EZH2	174 ± 84 nM	No	Preclinical	[63]
EPZ031686	SMYD3	36 nM	Yes	Preclinical	[64]
LLY-507	SMYD2	0.6 μM	No	Biological testing	[65]
A-893	SMYD2	42% reduction of p53K370me1	No	Biological testing	[66]

*Note*: KMT, lysine methyltransferase; EHMT1/2, euchromatic histone-lysine *N*-methyltransferase 1/2; EZH2, enhancer of zeste homologue 2; SMYD2/3, SET and MYND domain containing 2/3.

As the delicate balance in the activity of KMT and KDMs serves to tightly regulate Lys methylation and maintain healthy homeostatic conditions, it is not surprising that a disruption can lead to various pathologies. Although there has been research connecting non-histone Lys methylation to tumorigenesis, less has been explored related to other pathological conditions, which presents a unique opportunity for future research. The Lys methylation of heat shock protein 70 (HSP70) plays a role in homeostasis and an over-abundance of HSP70 has been found to be implicated in autoimmune diseases [49]. For example, elevated level of HSP70 have been found in preeclampsia patients and is thought to contribute to the oxidative stress and inflammation that is characteristic of this condition [50]. HSP70 is also subjected to methylation at K561 and the presence of methylation site hints at a possible regulatory role in HSP function [51].

To date, the focus of non-histone Lys methylation has been toward its role in human pathology, however, recent research has suggested that non-histone Lys methylation may also be functionally significant in plant cells [52]. Uncovering plant methyllysine proteome is still in its infancy. Lys methylated proteins have been discovered in cytochrome C in wheat and cauliflower [53], as well as in spinach calmodulin [54]. In addition, Lys methylation occurs on the large subunit of Rubisco from pea plants, tomato, and tobacco plants [55]. Over 30 KMTs in the seven-beta-strand (SBS) and SET domain families are estimated to be implicated in plant Lys methylation. Although a functional role for these Lys methylation events in plants are yet to be characterized, the impact of environmental stressors on the expression of genes encoding KMTs points to methylation being implicated in a protective response for plants [52]. As the emerging scope of Lys methylation is expanding, it is expected that ongoing research will continue to demonstrate far greater importance than was first surmised by the earliest discoveries.

## Summary

Decades of research into PTMs has characterized the functional importance of processes such as phosphorylation, acetylation, ubiquitination, and methylation of histones. Among these PTMs is a long-neglected and now rapidly expanding field of non-histone protein Lys methylation. Thanks to the advent of technologies, now we are able to perform molecular dynamics studies on KMT-catalyzed methylation of histone peptides that contain Lys and its sterically demanding analogs with mass spectrometry and nuclear magnetic resonance (NMR) spectroscopy [56]. Furthermore, PTM crosstalk between methylation and phosphorylation on histone peptides can be studied by host-assisted capillary electrophoresis. This is an effective method for studying PTM crosstalk with fast separation, high resolution, and low sample consumption [57]. However, there still remain a number of yet undiscovered Lys methylation sites within the proteome, and implications of these methylation events are still unclear. How dysfunction of Lys methylation contributes to carcinogenesis and how this intriguing PTM drives normal cell biology are looming questions within this relatively young research field and are intriguing questions of yet further study.

## Competing interests

The authors have declared no competing interests.
